# 

**Published:** 1908-05-15

**Authors:** 


### ANNOUNCEMENTS.

CLINICIANS FOR THE INDIANA STATE DENTAL MEETING, June 4th to 6th, 1908. Dr. Truman W. Brophy, Chicago, Ills., "Cleft Palate Operation." Dr. F. B. Morehead, Chicago, Ills., "Surgical Clinic." Dr. J. D. Patterson, Kansas City, Mo., "Pyorrhoea." Dr. E. H. Allen, Freeport, Ills., "Gold Filling." Dr. C. C. Corbett, Edwardsville, Ills., "Approximal Gold Filling with Cement Lining." Dr. J. K. Conroy, Belleville, Ills., "Gold Fillings using Non-Cohesive Gold in Gingival Third." Dr. William Finm, Cedar Rapids, Iowa, "Gold Filling in Approximo Occlusal Surface of an Upper Bicuspid." Dr. F. G. Richardson, Mason City, Iowa, "Gold Filling in Approximo Incisal of an Upper Anterior tooth." Dr. N. S. Hoff, Ann Arbor, Mich., "Treatment of Pyorrhoea." Dr. J. P. Buckley, Chicago, Ills., "Surgery of, and Drugs used in Pyorrhoea." Dr. M. H. Fletcher, Cincinnati, Ohio, "Pyorrhoea." Dr. W. F. Lowrenz, St. Louis, Mo., "Gold Inlay." Dr. F. H. Swartz, Morris, Ills., "Cast Gold Inlays." Dr. F. W. Williard, Anna, Ills., "Gold Inlay." Dr. W. H. Taggart, Chicago, Ills., "Cast Inlays and Bridges." Dr. Louis E. Bake, Chicago, Ills., "Hollow Cast Inlay." Dr. H. B. Tileston, Louisville, Ky., "Cast Gold Inlay." Dr. L. A. King, Henderson, Ky., "Cast Gold Inlays." Dr. Burton Lee Thorpe, St. Louis, Mo., "Gold Inlay." Dr. L. E. Custer, Dayton, Ohio, "Electrical Casting of Gold Inlay." Dr. Henry Barnes, Cleveland, Ohio, "Gold Inlay, using Platinum and Pure Gold." Dr. F. M. Fulkerson, Sedalia, Mo., "Cast Gold Inlay, using Hand Pressure." Dr. L. P. Davis, Lincoln, Neb., "Veneer Gold Inlay." Dr. Robert Seymour, Philadelphia, Pa., "Cast Gold Inlay." Dr. Fred McIntosh, Bloomington, Ills., "Forming Wax for Cast Gold Work, Investing of Same and Casting." Dr. Lee K. Stewart, Chicago, Ills., "Gold Inlay from Model." Dr. George C.

McCann, Danville, Ills., "Special Anterior Bridge Abutments and Variations of the same, for Permanent Splinting of Loose Teeth." Dr. G. W. Schwartz, Chicago, Ills., "Splint for Loosened Teeth." Dr. F. E. Roach, Chicago, Ills., "Cast Abutments for Bridge Work." Dr. W. M. McCall, Louisville, Ky., "Cast Abutments for Bridges." Dr. Max. M. Eble, Louisville, Ky., "Cast Attachments for Bridges." Dr. C. E. Bellchamber, Effingham, Ills., "Filling of Ascher's Artificial Enamel." Dr. C. M. Baldwin, Chicago, Ills., "Ascher's Artificial Enamel." Dr. Burton Lee Thorpe, St. Louis, Mo., "Translux Enamel Filling." Dr. H. H. Harrison, Wheeling, W. Va., "Ascher's Artificial Enamel." Dr. L. H. Arnold, Chicago, Ills., "Making of an all Carved Porcelain Jacket Crown." Dr. F. L. Wright, Wheeling, W. Va., "Porcelain Restoration." Dr. Alden Bush, Columbus, Ohio, "Manipulation of Porcelain." Dr. A. L. Le Gro, Detroit, Mich., "Labial Porcelain Restoration in Anterior Teeth." Dr. W. H. Cudworth, Milwaukee, Wis., "Porcelain Restoration." Dr. C. I. Keely, Hamilton, Ohio, "Cavity Preparation for Gold Inlays." Dr. Fred W. Gethro, Chicago, Ills., "Cavity Preparation for Gold Filling." Dr. Raymond E. Grant, Louisville, Ky., "Preparation of Cavities for Gold Inlays." Dr. Henry Pirtle, Louisville, Ky., "Combination Fillings, Cement and Amalgam." Dr. L. P. Bethel, Columbus, Ohio, "Little Helps in Orthontia." Dr. C. L. Snyder, Freeport, Ills., "Method of Anchoring Bridges Adapted to Cases where Lower Incisors have been Lost through Absorption." Dr. George W. Haskins, Chicago, Ills., "Telescoping Attachments for Partial Dentures." Dr. C. J. Lyons, Jackson, Mich., "Porcelain Crowns with Cast Cap and Dowel for Badly Decayed Roots." Dr. W. G. Bow, Louisville, Ky., "Restoration Badly Broken Down or Fractured Roots with Porcelain Crowns." Dr. C. E. Byington, Harrisburg, Ills., "A Time Saving Method in Constructing Shell Crowns." Dr. Walter Dittmar, Chicago, Ills., "Technique of an Accurate Method for making Gold Shell Crowns of Proper Contour." Dr. L. P. Davis, Lincoln, Neb., "Gold Crowns." Dr. Willis

Coston, Topeka, Kan., "Gold and Porcelain Bridge Work." Dr. Harry Lee, Louisville, Ky., "Bridge Work." Dr. M. H. McMillan, Roseville, Ills., "Some Useful Methods." Dr. E. B. Spalding, Detroit, Mich., "Splint for Teeth when Impossible to Set Posts Parallel." Dr. William H. DeFord, DesMoines, Iowa, "Instructions in Somnoform Administration." Dr. George W. Schwartz, Chicago, Ills., "Removable Bridge Work." Dr. J. H. Prothero, Chicago, Ills., "Anatomic Occlusion of Artificial Teeth." Dr. George H. Wilson, Cleveland, Ohio, "Antagonizing Complete Artificial Dentures." Dr. E. J. Perry, Chicago, Ills., "Anatomic Occlusion of Artificial Teeth." Dr. Robert Canine, Louisville, Ky., "Preparation of Cast for Temporary Teeth." Dr. Walter Dittmar, Chicago, Ills., "Exhibit of Natural Sized Models of Teeth, Tooth Dissections, Drawings and Measurements, showing the Contour of Normal Shaped Crowns.

—✦—

MEETING OF THE OHIO STATE DENTAL BOARD:—The regular semi-annual meeting of the State Dental Board of Ohio will be held in Columbus, June 16, 17 and 18, 1908, at the Bliss Business College, 185½ S. High Street.

At the present time the Board cannot accept a license from the Board of Examiners of another state. Applications must be accompanied by the fee of twenty-five dollars ($25) ($25.00), and should be in the hands of the Secretary not later than June 6th.

For further information and application blanks, address the Secretary.

F. R. CHAPMAN, D. D. S., *Secy.*
305 Schultz Bldg.,
Columbus, Ohio.

